# The relationships between teachers’ emotional labor and display rules, trait emotions, exhaustion, and classroom emotional climate

**DOI:** 10.3389/fpsyg.2023.957856

**Published:** 2023-02-27

**Authors:** Pei Ma, Lichang Zhang, Hui Dong, Jian Yu

**Affiliations:** ^1^Faculty of Education, Shaanxi Normal University, Xi'an, China; ^2^No. 82 Middle School, Zhengzhou, China; ^3^College of Teacher Education, Hebei Normal University, Shijiazhuang, China

**Keywords:** teachers’ emotional labor strategies, classroom setting, display rules, trait emotions, classroom emotional climate, emotional exhaustion

## Abstract

**Introduction:**

Emotions are an integral part of education, and the way teachers manage their emotions is crucial to educational success. This study focuses on teachers’ emotional labor in secondary school classrooms and examined the relationships between emotional labor strategies and display rules, trait emotions, emotional exhaustion, and classroom emotional climate.

**Methods:**

In the study, 496 secondary school teachers (386 female) aged 21–59 years (mean age = 37.61 ± 8.87 years) completed five self-reported questionnaires. Data were analyzed using structural equation model in AMOS.

**Results:**

The results showed that (1) display rules provide positive situations to deep acting and the expression of naturally felt emotions and mediate teachers’ positive emotions and strategies; (2) positive trait emotions increase the expression of naturally felt emotions and negative trait emotions increase surface acting; (3) surface acting results in emotional exhaustion and has an adverse impact on classroom emotional climate; and (4) deep acting and the expression of naturally felt emotions positively affect classroom emotional climate.

**Discussion:**

These findings revealed that deep acting and the expression of naturally felt emotions are positively related to positive emotions and the classroom setting, whereas surface acting plays a negative role in the emotional states of individuals and the classroom. The study gives the centrality of teacher emotions in the teaching and learning process, clarifies some antecedents and consequences related to emotional labor strategies in a classroom setting, and provides some ideas to optimize educational outcomes. The five variables presented in the study are good examples that can contribute to protecting teachers’ wellbeing and improving the psychosociological environment.

## 1. Introduction

Teaching is an emotional practice (Hargreaves, 2001; Agudo, 2018). In classrooms, showing positive and concealing negative emotions is encouraged to benefit students by creating an appropriate atmosphere (Shewark et al., 2018; Santos et al., 2021), predicting good academic outcomes (Nalipay et al., 2019), and setting preferable examples for improving students’ social competence (Schutz and Pekrun, 2007; Xie et al., 2022). However, presenting the required emotions and hiding negative ones may cause strain on teachers (Taxer and Frenzel, 2018; Lee, 2019). To reveal the emotional efforts that teachers take, teachers’ emotional labor (TEL) has been studied (Hargreaves, 2000; Yin et al., 2017; Li and Liu, 2021; Zhu et al., 2021). In the interaction with students, colleagues, school administrators, parents, and others around them, teachers inhibit, generate, and manage their feelings and expressions of emotions consistent with certain institutional norms (Hargreaves, 2000; Yin et al., 2017). In the past three decades, studies have clarified its vital roles in both achieving educational goals (Yin and Lee, 2012; Burić and Frenzel, 2021; Li and Liu, 2021) and monitoring teachers’ emotional state and regulation (Noor and Zainuddin, 2011; Yao et al., 2015; Lee et al., 2016). Some strategies such as surface acting, deep acting, and the expression of naturally felt emotions have been verified in teachers’ emotional labor (Yin, 2012; Wang et al., 2020; Zhu et al., 2021). Moreover, some antecedents (e.g., emotional intelligence) and consequences (e.g., job satisfaction of TEL have been explored Zhang and Zhu, 2008; Noor and Zainuddin, 2011; Yin et al., 2013; Han et al., 2021). However, these studies addressed related factors of TEL in generic professional settings. Research on TEL in a classroom setting—teachers’ core professional workplace—is limited.

The classroom, where secondary school students and teachers spend most of their time, is an emotional setting (Agudo, 2018). TEL in the classroom implies teachers’ cognition of the teacher–student relationship and teaching (Hargreaves, 2000) and influences the teaching process and students’ learning (Isenbarger and Zembylas, 2006; Keller et al., 2014). Although TEL in classrooms is a part of teachers’ broader emotional labor, there still exist some differences. First, as the “working environment does influence required emotions” (Grandey and Gabriel, 2015, p. 328), teachers deal with their emotions in accordance with the emotional requirements of classrooms that require teachers to maintain positive emotions, repress negative emotions, and use emotions as a teaching tool (Yin and Lee, 2012; Waldbuesser et al., 2021), which endows TEL with more pedagogical features. Second, the teacher–student interaction is the main interactional form in classrooms (Zembylas, 2005) where emotion regulation occurs to deal with teachers’ internal conflicts (e.g., between teaching beliefs and curriculum requirements) and those between teachers and students (Loh and Liew, 2016; Miller and Gkonou, 2018), rather than conflicts with people outside classrooms. Third, in classrooms, teachers’ emotional performance tends to be restrained. For instance, by sharing fewer emotions (Isenbarger and Zembylas, 2006) and inhibiting their emotions temporarily (Gregersen, 2007), some teachers can keep their “micro-politically superior position” (Hargreaves, 2000, p. 819) and concentrate students and themselves on teaching. As emotional requirements, emotion regulation and performance can impact how individuals use emotional labor strategies (Brotheridge and Lee, 2003; Grandey and Gabriel, 2015), TEL in classrooms may have its own features. Moreover, during the COVID-19 pandemic, online teaching is frequent, which extended classrooms to teachers’ private life and heightened teachers’ workloads (Ghasemi, 2022) with greater emotional stress (Carroll et al., 2022). Therefore, it is necessary to explore the mechanism of TEL in classrooms. However, in previous studies, the classroom is rarely treated as an independent professional setting. The existing research on TEL in teaching mainly describes and explains the functions and forms (Yin and Lee, 2012; Loh and Liew, 2016; Miller and Gkonou, 2018; Nyanjom and Naylor, 2021), and investigations on strategies and related factors of TEL in classrooms are limited.

To overcome the limitations, this study centered TEL in the teaching and learning process and constructed a structural equation modeling to explore the mechanism of TEL in classrooms. Specifically, the relationships between TEL strategies, display rules, trait emotions, emotional exhaustion, and classroom emotional climate were tested through Grandey’s integrative model of emotional labor (Grandey, 2000), which can “better control for potential overlap” of the indicators related to emotional labor (Hall, 2019, p. 366), provide some evidence on the way that teachers deal with their emotions, how TEL is influenced and how it contributes to both teachers’ wellbeing and classrooms, and can assist to improve TEL in classroom settings.

### 1.1. Grandey’s integrative model of emotional labor

Grandey’s integrative model of emotional labor (Grandey, 2000) was proposed to present emotional labor and its related factors. In this model, emotional labor is defined as “the process of regulating both feelings and expressions for the organizational goals” (Grandey, 2000, p. 97). In the center of the model, emotional labor strategies are explained with emotional regulation theory. It argues that when the felt emotions are inconsistent with the required emotions, response-focused regulation corresponds with the process of surface acting in which individuals change their observable expressions to correspond to the situation; however, the emotion is not felt. The antecedent-focused emotion regulation corresponds with the process of deep acting in which one modifies how he or she perceives the situation with cognitive techniques to display the required emotions (Grandey, 2000; Yin et al., 2017). However, in this model, the expression of naturally felt emotions in which felt emotions coincide with required emotions and individuals consciously express the felt emotions (Diefendorff et al., 2005) is not involved.

Around the strategies, indicators related to emotional labor are presented, which consist of situational cues, individual factors, organizational factors, consequences to individual wellbeing, and organizational performance. Situational cues contain the interaction expectations and emotional events. As the chronic situational factor, the interaction expectations vary in different work contexts and are controlled by the display rules of the organization. Emotional events at work have an immediate impact on emotional labor. Grandey (2000) concluded with four individual factors: sex, emotional expressivity, emotional intelligence, and affectivity and three organizational factors: job autonomy, supervisor, and co-worker support. The consequences of emotional labor are classified into two aspects: individual wellbeing (e.g., burnout and job satisfaction) and organizational performance (e.g., performance and withdrawal behaviors). As the model summarizes the variables as antecedents and consequences of emotional labor and focuses both on employees and organizations, it has been used to uncover the mechanism of emotional labor (Yin et al., 2017; Han et al., 2021). For instance, to explore the relationships between college TEL and emotional job demands, teaching support, and teacher efficacy, the model is applied and confirmed to be efficient (Han et al., 2021). The model also explained the mechanism of TEL in kindergarten teachers whose emotional labor can predict job satisfaction and be impacted by instructional leadership and trust in colleagues (Yin et al., 2022) and can be significantly related to the sense of efficacy and children’s social–emotional development and learning (Xie et al., 2022). In addition, variables of culture (e.g., Confucian familism) and family (e.g., work–family conflict) to TEL were tested in the model (Zhu et al., 2021).

In the past two decades, several relationships in the model have been confirmed through these studies (Yin et al., 2017; Han et al., 2021; Zhu et al., 2021; Xie et al., 2022; Yin et al., 2022), thereby inspiring this research to examine the model in the secondary school classroom where the work context is at the micro-level and TEL could differ. In addition, it should be noted that this study only examined five related variables, and the results could not represent the whole framework.

### 1.2. Display rules and their relationship with TEL strategies in classrooms

*Display rules* are a term used in emotional labor that describes the organizational norms regarding “whether expression of certain emotions is acceptable within the context of one’s professional work” (Stark and Bettini, 2021, p. 2). In emotional labor, display rules can reflect the cultural, social, and professional expectations and norms of an organization (Yin and Lee, 2012), guide employees in “establishing the sense of entitlement or obligation that governs emotional exchanges” (Hochschild, 1983, p. 56), and drive them to apply emotional labor strategies (Stark and Bettini, 2021). For example, Kammeyer-Mueller et al. (2013) found that perceived display rules are positively related to the frequency of emotional labor. Other studies have indicated that display rules can predict surface acting and deep acting (Trougakos et al., 2011).

Since how employees identify with job role can impact their use of strategies (Schaubroeck and Jones, 2000) and in classrooms, teachers verify their identity as learning supporters that prevails over others (De Costa et al., 2018); TEL can be regarded as the means to support students’ emotional and academic development (Miller and Gkonou, 2018; Stark and Bettini, 2021). In addition to hiding negative emotions and maintaining positive emotions, teachers instrumentalize emotions to achieve teaching goals in classroom teaching (Yin and Lee, 2012; Waldbuesser et al., 2021). In online classes, the rules still appropriate that TEL is used to ensure effective teaching and maintaining connections with students (Nyanjom and Naylor, 2021). However, these studies have not regarded display rules as an indicator to investigate the impact of display rules on TEL strategies in classrooms.

This study adopted the view that display rules can be measured by the frequency, intensity, and variety of emotional labor (Brotheridge and Lee, 2003; Grandey and Melloy, 2017) and focused on classroom settings to explore the relationships between them. Based on the previous findings, we proposed Hypothesis 1:

Display rules are positively related to surface acting (H1a), deep acting (H1b), and the expression of naturally felt emotions (H1c).

### 1.3. Teachers’ trait emotions and their relationship with TEL strategies in classrooms

Teachers experience various emotions while working. These feelings can be categorized using state and trait levels. The state emotions are relatively short episodes, and the trait emotions are “individual dispositions to frequently experience emotions of a given kind” (Burić et al., 2017, p. 326). In previous studies that explain teachers’ professional competence and examine the influence of emotions on their work performance (Burić et al., 2019; Nalipay et al., 2019), teachers’ trait emotions have been used more as trait emotions are more stable and profession-linked.

Researchers have found that teachers’ feelings toward teaching can inform what strategies can be used and how they can be applied in teaching (Zembylas, 2005; Lee, 2019; Wang et al., 2020). Previous studies have explored the relationships between teachers’ trait emotions and TEL, showing that enjoyment and love were positively related to deep acting and negatively related to surface acting, while negative emotions (e.g., anxiety and anger) were positively related to surface acting (Lee et al., 2016; Burić et al., 2019). In classrooms, as negative emotions can be used strategically for classroom management (Wang et al., 2020) and teachers need not interact with people outside classrooms, the relationship may differ. In addition, the relationship between teachers’ trait emotions and the expression of naturally felt emotions in teaching is seldom tested. Therefore, this study explored how teachers’ trait emotions impact their application of emotional labor strategies in classrooms. Based on the previous findings and the view that expressing felt positive emotions is encouraged in teaching, this study proposed Hypothesis 2:

Teachers’ negative trait emotions are positively related to surface acting (H2a), and teachers’ positive trait emotions are negatively related to surface acting (H2b), positively related to deep acting (H2c) and the expression of naturally felt emotions (H2d).

Furthermore, in primary and secondary classroom contexts, teachers use emotions to maintain their “micro politically superior position” (Hargreaves, 2000, p. 819). Display rules are associated with politics, society, and culture (Hargreaves, 2001; Zembylas, 2005). Therefore, in classrooms, teachers have a high degree of autonomy in perceiving display rules. As some traits of an individual may be related to emotional requirements (Dahling and Johnson, 2013), this study hypothesized that teachers’ trait emotions may affect their preservation of display rules, and two hypotheses were proposed:

Teachers’ negative emotions are negatively related to display rules (H2e), and positive emotions are positively related to display rules (H2f).

### 1.4. TEL strategies in classrooms and their relationship with emotional exhaustion

Emotional exhaustion, referring to the “feelings of being overextended and depleted of one’s emotional and physical resources” (Maslach et al., 2001, p. 399), is an index of individual wellbeing caused by emotional labor. In explaining the relationship between TEL strategies and an individual’s emotional exhaustion, the conservation of resources theory was adopted, which argues that emotional labor strategies can gain, conserve, and use resources (Brotheridge and Lee, 2002). If TEL is effective (e.g., deep acting and the expression of naturally felt emotions), the psychological resources could be maintained or increased; thus, job satisfaction improves (Huang et al., 2019). Contrastingly, if it is ineffective (e.g., surface acting), teachers may have emotional dissonance (Hülsheger and Schewe, 2011) and resource loss can be caused. Once resources are excessively consumed without timely replenishment, one may experience emotional exhaustion (Keller et al., 2014; Yao et al., 2015). Previous studies have tested the relationships and found that surface acting could result in teachers’ emotional exhaustion (Zhang and Zhu, 2008; Näring et al., 2012; Zhu et al., 2021), while the relationships with deep acting and the expression of naturally felt emotions were complicated. Deep acting was negatively related (Yao et al., 2015), positively related (Zhu et al., 2021), or not related (Näring et al., 2012) to emotional exhaustion. The expression of naturally felt emotions had no relationship (Näring et al., 2012) or a negative relationship (Zhu et al., 2021) with emotional exhaustion.

In the classroom, teaching time is limited, and achieving teaching goals is the focus. The resource consumption in TEL can be different. For instance, teachers may transform their focus on teaching to reduce consumption, the requirement that teachers cannot leave classrooms when they feel stressed during teaching time may increase consumption, and achieving teaching goals well and receiving students’ care may supply resources (Duke et al., 2009). To reveal how TEL strategies consume the resources in the classroom setting, the present study proposed Hypothesis 3:

Surface acting (H3a) and deep acting (H3b) are positively related to emotional exhaustion, and the expression of naturally felt emotions is negatively related to emotional exhaustion (H3c).

### 1.5. TEL strategies in classrooms and their relationship with classroom emotional climate

Grandey and Melloy (2017) argued that emotional labor affects emotional performance in an organization, which affects the organization’s wellbeing. In this study, the classroom is the micro-level organization of a school, and teachers’ preservation of the classroom’s emotional climate can be regarded as the organizational consequence. The classroom emotional climate is the psychosociological environment that is created by “the quality of social and emotional interactions in the classrooms between and among students and teachers” (Reyes et al., 2012, p. 700), which can be influenced by TEL. On the one hand, teachers regard their emotions as prominent determinants of classroom emotional climate (Shewark et al., 2018) and apply strategies to maintain a positive and well-managed classroom emotional climate to benefit students. On the other hand, teachers’ display of beneficial emotions can inspire productive teacher–student interactions and encourage students’ engagement in learning activities (Taxer and Frenzel, 2018), which can assist to form a better classroom emotional climate.

As students can detect teachers’ feelings (Xu, 2018) and emotions can be transmitted among students and teachers (Frenzel et al., 2018), deep acting and the expression of naturally felt emotions can be treated as beneficial since the display of emotions is genuine. In contrast, surface acting may cause emotional misunderstanding in classroom interactions (Grandey and Melloy, 2017; Yin et al., 2017). Therefore, the study proposed Hypothesis 4:

Surface acting is negatively related to the teacher-perceived classroom emotional climate (H4a), while deep acting (H4b) and the expression of naturally felt emotions (H4c) are positively related to the teacher-perceived classroom emotional climate.

### 1.6. This study

In brief, the essential role of TEL in the teaching and learning process (Isenbarger and Zembylas, 2006; Yin and Lee, 2012; Burić and Frenzel, 2021) and the differences between TEL in a classroom setting and their broader professional settings (Loh and Liew, 2016; Miller and Gkonou, 2018; Waldbuesser et al., 2021) make it necessary to explore the relationships between emotional labor strategies and the related factors. To clarify the gap in TEL and class teaching (Grandey, 2000; Stark and Bettini, 2021) and enhance the professionalization of teachers in 21st-century classrooms where emotions are associated with students’ outcomes (Taxer and Frenzel, 2018), this study adopts Grandey’s integrative model of emotional labor to examine one possible mechanism of TEL in classrooms, where the TEL strategies are related to indicators of display rules as a situation cue, teachers’ trait emotions as an individual factor, emotional exhaustion as an individual consequence, and classroom emotional climate as an organizational consequence. Therefore, the research question is proposed: what are the relationships between TEL strategies in classrooms and display rules, teachers’ trait emotions, emotional exhaustion, and classroom emotional climate? To answer this question, four hypotheses are raised.

*Hypothesis 1*: Display rules are positively related to surface acting (H1a), deep acting (H1b), and the expression of naturally felt emotions (H1c).

*Hypothesis 2*: Teachers’ negative trait emotions are positively related to surface acting (H2a), and teachers’ positive trait emotions are negatively related to surface acting (H2b), positively related to deep acting (H2c) and the expression of naturally felt emotions (H2d). Teachers’ negative emotions are negatively related to display rules (H2e), and positive emotions are positively related to display rules (H2f).

*Hypothesis 3*: Surface acting (H3a) and deep acting (H3b) are positively related to emotional exhaustion, and the expression of naturally felt emotions is negatively related to emotional exhaustion (H3c).

*Hypothesis 4*: Surface acting is negatively related to the teacher-perceived classroom emotional climate (H4a), while deep acting (H4b) and the expression of naturally felt emotions (H4c) are positively related to the teacher-perceived classroom emotional climate.

**Figure 1:**
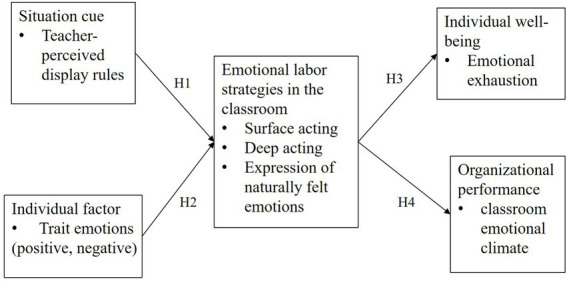
Hypothesis model of this study.

To intuitively present these hypotheses in Grandey’s integrative model of emotional labor, the hypothesized model for testing is shown in Figure 1.

## 2. Methods

### 2.1. Participants

A total of 496 secondary school teachers participated in the investigation, which fit the criteria of Barrett (2007); that is, the sample should be greater than 10 times the number of variables and between 200 and 500, as the chi-square value of the maximum likelihood method will significantly expand, resulting in poor model fitting degree (if the sample was over 500). The sample comprised 386 women and 110 men whose ages ranged from 21 to 59 years (mean age = 37.61 ± 8.87 years) and whose years of teaching ranged from 0 to 38 years (mean = 14.71 ± 10.08 years). Participants covered all the subjects of Chinese secondary schools and were from 18 provinces, municipalities, and autonomous regions of China.

### 2.2. Measures

Five self-report questionnaires were used to test eight variables in this study. We invited two language teachers to translate and modify the content of items to ensure that the language was concise and precise. In addition, an expert panel was formed comprising seven professionals (three secondary school teachers, two consultants in educational statistics, one professor in education, and one teaching investigator from the teaching research office) to assess the items.

Teachers’ emotional labor strategy in the classroom was measured by the *Teachers’ Emotional Labor Strategy Scale* (Yin, 2012). With a few items revised to fit the classroom setting, the 13-item scale (Appendix A) consists of three dimensions: surface acting, four items (*α* = 0.85; e.g., *I just pretend to have the emotions I need to display in class*); deep acting, five items (*α* = 0.88; e.g., *I make an effort to actually experience the emotions that I need to display during class teaching*); and expression of naturally felt emotions, four items (*α* = 0.85; e.g., *The emotions I show to students in class correspond with what I feel spontaneously*). All the items were scored on a five-point Likert scale ranging from 1 (strongly disagree) to 5 (strongly agree), and the internal consistency analysis showed a Cronbach’s alpha of 0.79 for the 13-item scale, indicating acceptable reliability.

Display rules in the classroom were measured by the emotion-related role requirements in the *Emotional Labour Scale* (Brotheridge and Lee, 2003), which has been used to measure display rules (Grandey and Melloy, 2017). With a few items revised to fit the classroom setting, the six-item scale consists of one dimension (e.g., *I display specific emotions required by the class teaching*), and all the items were scored on a five-point Likert scale ranging from 1 (*strongly disagree*) to 5 (*strongly agree*). The internal consistency analysis showed a Cronbach’s alpha of 0.87, indicating good reliability.

Teachers’ trait emotions were measured using a revised *Teacher Emotion Questionnaire* (Burić et al., 2017), and the answers were obtained according to a five-point Likert scale, ranging from 1 (*strongly disagree*) to 5 (*strongly agree*). The nine-item scale consisted of two dimensions: positive trait emotions, five items (*α* = 0.94, e.g., *I am happy when students understand the materials*); and negative emotions, four items (*α* = 0.82, e.g., *While working with completely unmotivated students, I feel there is no way out*). All the items were scored on a five-point Likert scale ranging from 1 (*strongly disagree*) to 5 (*strongly agree*), and the internal consistency analysis showed a Cronbach’s alpha of 0.79 for the nine-item scale, indicating acceptable reliability.

Classroom emotional climate was measured using a unidimensional six-item questionnaire (Appendix B) developed for this study, referring to both *The School-Level Environment Questionnaire* (Johnson et al., 2007) and five emotional elements of classroom emotional climate (emotional awareness, relationships, management, and interpersonal beliefs and guidelines) proposed by Harvey et al. (2012). All six items (e.g., *Most students react to my emotions*) were scored on a five-point Likert scale ranging from 1 (*strongly disagree*) to 5 (*strongly agree*), and the internal consistency analysis showed a Cronbach’s alpha of 0.83, indicating good reliability.

Emotional exhaustion was measured by the emotional exhaustion aspect of the *Chinese Primary and Secondary School Teachers’ Job Burnout Questionnaire* (Wu et al., 2016), a Chinese revision of the Maslach Burnout Inventory Education Survey. All eight items (e.g., *I feel that teaching has drained me emotionally*) were scored on a five-point Likert scale ranging from 1 (*strongly disagree*) to 5 (*strongly agree*), and the internal consistency analysis showed a Cronbach’s alpha of 0.93, indicating excellent reliability.

### 2.3. Procedures

A strict procedure was followed in this study. First, 20 teachers and headmasters from urban and rural schools in different provinces were encouraged to ask questions to ensure the validity of the pretest. Then, the study was approved by the Institutional Review Board of the Faculty of Education in our university (No. 2019TS054). Second, the study was conducted according to the Chinese Survey and Behavioral Research Ethics Requirements. Prior to the investigation, informed written consent was obtained from all participants who were informed about the nature and goals of the study, anonymity of the data collection, instructions on how to complete the questionnaires, and their rights to withdraw from the study at any time. Third, data collection and analyses followed the Rules of Academic Ethics described by the Chinese Academy of Social Sciences. Owing to the COVID-19 pandemic, data were collected online from 2 January 2022 to 28 January 2022, with the assistance of teachers and headmasters in pretests. IBM Statistical Package for Social Sciences (SPSS) 26.0 and IBM AMOS 23.0 were used for data analyses.

### 2.4. Data analysis

A series of confirmatory factor analyses were used to test the construct validity of the five questionnaires separately and the single-factor model for analyzing the common method bias in AMOS 23.0. An exploratory factor analysis of the TEL strategies scale in the classroom was conducted to detect common method bias. Cronbach’s alpha for the subscales’ reliability and the descriptive statistics and correlations among latent variables were calculated using SPSS 26.0. Confirmatory factor analyses were performed to confirm the measurement model and structural equation modeling using AMOS 23.0. In assessing a structural equation model, fit indicators of *χ^2^*/df, comparative fit index (CFI), Tucker–Lewis index (TLI), and root mean square error of approximation (RMSEA) are recommended by Schreiber et al. (2006). Hu and Bentler (1999) examined the adequacy of some fit indexes and proposed that it is acceptable for 3 < *χ^2^*/df < 5, CFI and TLI values of >0.90, and RMSEA of <0.06. The values of CFI and TLI more than 0.95 and an RMSEA less than 0.05 can be good (Byrne, 2016), and “less than 0.08 corresponds to an acceptable fit” (McDonald and Ho, 2002, p. 72).

In addition, as the sample size is under 500, the mediation effect was tested by the method of bootstrapping with 5,000 samples. Bootstrap sampling is a type of resampling in which a large number of smaller samples of the same size are repeatedly taken, with replacement from the data set (Preacher and Hayes, 2008).

### 2.5. Construct validity and reliability

Table 1 reports the construct validity and reliability of the questionnaires. Cronbach’s alpha values for all variables ranged from 0.82 to 0.94, indicating adequate reliability.

**Table 1:** Latent variables, descriptive statistics, and reliability.

Variable	No. of items	Cronbach’s *α*	*χ^2^*/df	CFI	TLI	RMSEA	SRMR
Display rules	6	0.87	3.42	0.98	0.97	0.070	0.027
Positive trait emotions	5	0.94	3.34	0.98	0.97	0.069	0.046
Negative trait emotions	4	0.82					
Surface acting	4	0.85	2.33	0.97	0.97	0.052	0.047
Deep acting	5	0.88					
Expression of naturally felt emotions	4	0.85					
Emotional exhaustion	8	0.93	6.76	0.96	0.94	0.108	0.041
Classroom emotional climate	6	0.83	4.39	0.97	0.95	0.083	0.034

The model fit indices of display rules scale (*χ^2^*/df = 3.42, *p* < 0.01, CFI = 0.98, TLI = 0.97, RMSEA = 0.070, SRMR = 0.027), teachers’ trait emotions scale (*χ^2^*/df = 3.34, *p* < 0.01, CFI = 0.98, TLI = 0.97, RMSEA = 0.069, SRMR = 0.046), and TEL strategy scale (*χ^2^*/df = 2.33, *p* < 0.01, CFI = 0.97, TLI = 0.97, RMSEA = 0.052, SRMR = 0.047) are all within the acceptable limits.

The chi-square (*χ^2^*/df = 6.76 > 5) and RMSEA (RMSEA = 0.108 > 0.08) of emotional exhaustion, RMSEA (RMSEA = 0.083 > 0.08) of classroom emotional climate are beyond the limits. Considering the other fit indices are good and the emotional exhaustion questionnaire of the Maslach Burnout Inventory Education Survey has been tested and applied in other studies, the validity of the two scales can be accepted and used in this study.

The measurement model was tested to examine the construct validity. The results showed that the measurement model had a good fit (*χ^2^* = 1551.551, df = 791, *χ^2^*/df = 1.962, *p* < 0.01, TLI = 0.934, CFI = 0.939, RMSEA = 0.044). The factor loadings ranged from 0.55 to 0.92, indicating that the items of all the latent variables were preferable.

As this study applied self-reported questionnaires, results may be plagued by the common method bias. Therefore, Harman’s single-factor test *via* a confirmatory factor analysis and an exploratory factor analysis was used (Podsakoff et al., 2003). In the confirmatory factor analysis, the single-factor model was rejected (*χ^2^* = 8982.741, df = 819, *χ^2^*/df = 10.968, *p* < 0.01, TLI = 0.311, CFI = 0.345, RMSEA = 0.142). In exploratory factor analysis, the maximum variance method was used to extract a common factor for all the items, and the results showed that a single factor could account for 25.7% of the total variance, which is lower than 40%. In comparison, the eight factors potentially account for 69.9% of the total variance. Both the confirmatory and exploratory factor analyses suggested that the single factor should be rejected, indicating that the results were not plagued by the common method variance (Yin et al., 2017).

## 3. Results

### 3.1. Descriptive statistics and correlations

Table 2 presents mean, standard deviation and Person correlations among the eight latent variables. Results showed that display rules were significantly correlated with deep acting (*β* = 0.60, *p* < 0.001, H1b), the expression of naturally felt emotions (*β* = 0.52, *p* < 0.001, H1c), and positive trait emotions (*β* = 0.49, *p* < 0.001, H2f). Display rules were not correlated with teachers’ negative trait emotions (*β* = 0.03, *p* = 0.45, H2e), surface acting (*β* = 0.08, *p* = 0.07, H1a), and emotional exhaustion (*β* = 0.02, *p* = 0.62). Teachers’ positive emotions were significantly correlated with deep acting (*β* = 0.40, *p* < 0.001, H2c) and the expression of naturally felt emotions (*β* = 0.38, *p* < 0.001, H2d) but not correlated with teachers’ negative trait emotions (*β* = 0.04, *p* = 0.37), surface acting (*β* = 0.01, *p* = 0.86, H2b), and emotional exhaustion (*β* = 0.03, *p* = 0.52). Negative trait emotions were positively correlated with surface acting (*β* = 0.25, *p* < 0.001, H2a) and emotional exhaustion (*β* = 0.53, *p* < 0.001) but not correlated with deep acting (*β* = 0.00, *p* = 0.99) and expression of naturally felt emotions (*β* = 0.03, *p* = 0.58). The three strategies were correlated (*β_Surface acting-Deep acting_* = 0.13, *p* < 0.01; *β_Surface acting-Expression of naturally felt emotions_* = −0.14, *p* < 0.01; *β_Deep acting-Expression of naturally felt emotions_* = 0.40, *p* < 0.001). Surface acting was significantly correlated with emotional exhaustion (*β* = 0.40, *p* < 0.001, H3a), while deep acting was neither correlated with emotional exhaustion (*β* = 0.00, *p* = 0.95, H3b) nor the expression of naturally felt emotions (*β* = −0.02, *p* = 0.65, H3c). Moreover, classroom emotional climate was positively correlated with display rules (*β* = 0.52, *p* < 0.001), positive trait emotions (*β* = 0.50, *p* < 0.001), deep acting (*β* = 0.37, *p* < 0.001, H4b), and the expression of naturally felt emotions (*β* = 0.41, *p* < 0.001, H4c) and negatively correlated with surface acting (*β* = −0.12, *p* < 0.01, H4a), negative trait emotions (*β* = −0.13, *p* < 0.01), and emotional exhaustion (*β* = −0.13, *p* < 0.01) (Table 2).

**Table 2:** Correlation matrix among the latent variables.

Variables	Display rules	Positive trait emotions	Negative trait emotions	Surface acting	Deep acting	Expression of naturally felt emotions	Emotional exhaustion	Classroom emotional climate
Display rules	1							
Positive trait emotions	0.49^***^	1						
Negative trait emotions	0.03	0.04	1					
Surface acting	0.08	0.01	0.25^***^	1				
Deep acting	0.60^***^	0.40^***^	0.00	0.13^**^	1			
Expression of naturally felt emotions	0.52^***^	0.38^***^	0.03	−0.14^**^	0.40^***^	1		
Emotional exhaustion	0.02	0.03	0.53^***^	0.40^***^	0.00	−0.02	1	
Classroom emotional climate	0.52^***^	0.50^***^	−0.13^**^	−0.12^**^	0.37^***^	0.41^***^	−0.13^**^	1
Mean	3.26	4.39	3.42	2.87	3.76	3.70	3.33	3.79
Standard deviation	0.54	0.71	0.77	0.94	0.77	0.70	0.90	0.53

^**^p < 0.01, ^***^p < 0.001.

### 3.2. Hypothesis test

**Figure 2:**
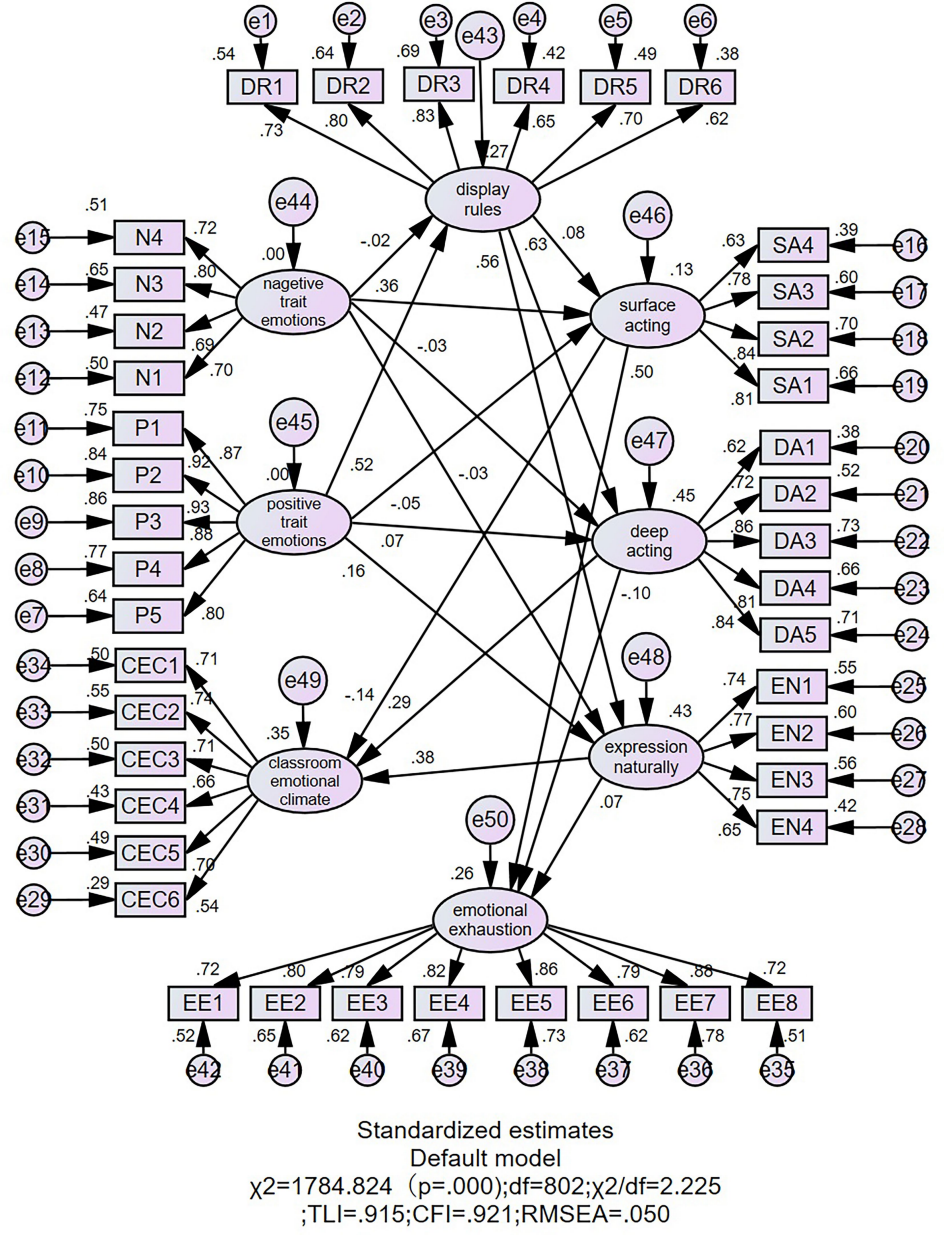
Structural equation model results (*N* = 496). Expression naturally = expression of naturally felt emotions (Full name is too long to be validated in AMOS).

The hypothesized model in Figure 2 displays the results of the structural equation model between display rules, teachers’ trait emotions, their emotional exhaustion, classroom emotional climate, and TEL in the classroom. The results showed that the model had a good fit (*χ^2^* = 1784.824, df = 802, *χ^2^*/df = 2.225, *p* < 0.01, CFI = 0.921, TLI = 0.915, RMSEA = 0.050).

**Figure 3:**
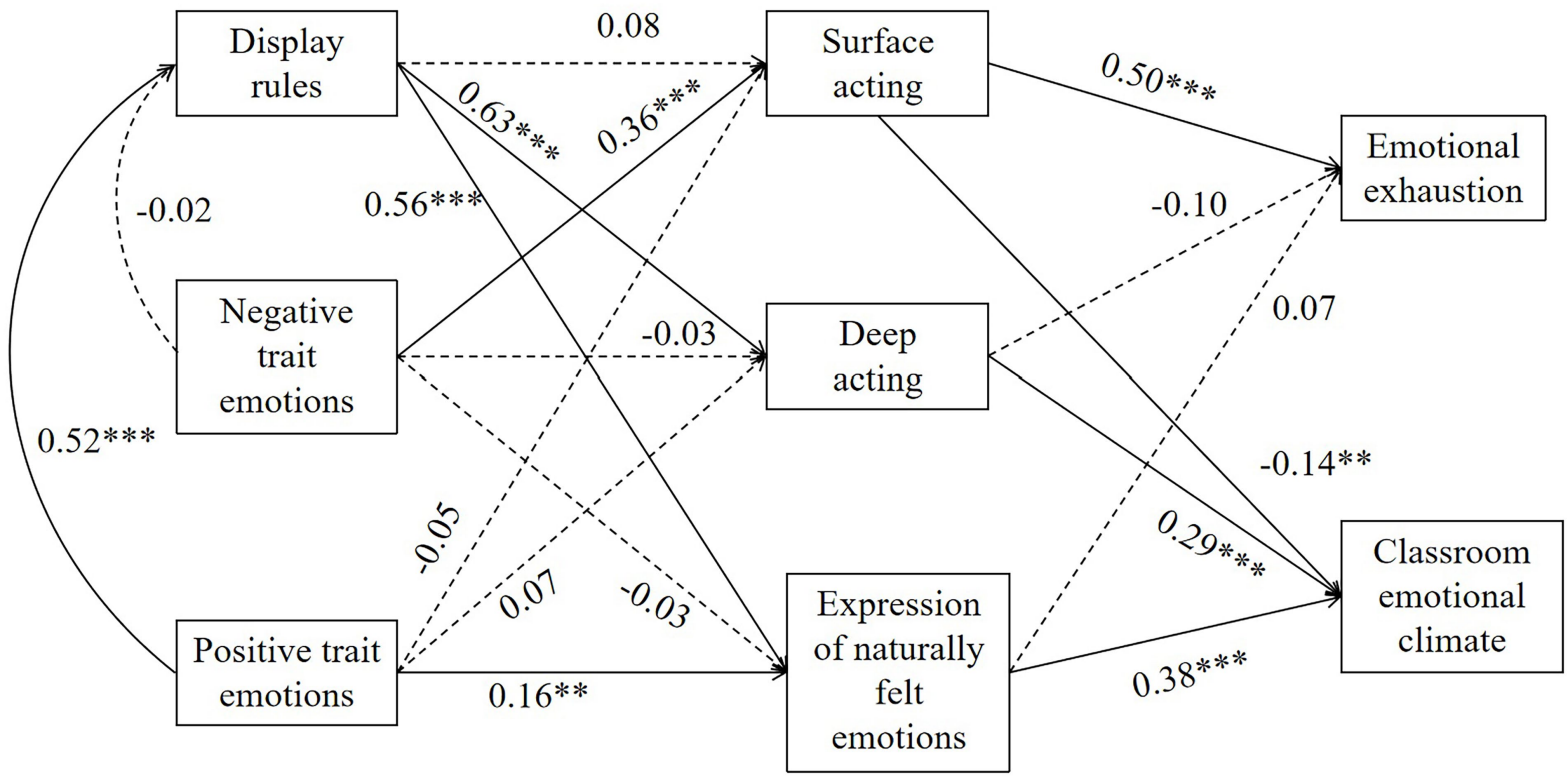
Schematic diagram of structural equation model results. **p < 0.01, ***p < 0.001.

Figure 3 presents the standardized regression weight and the significance concisely.

As presented in Figure 2 and Figure 3, display rules were positively related to deep acting (*β* = 0.63, *p* < 0.001, H1b) and the expression of naturally felt emotions (*β* = 0.56, *p* < 0.001, H1c) but not significantly correlated with surface acting (*β* = 0.08, *p* = 0.15, H1a). Teachers’ negative trait emotions were positively related to surface acting (*β* = 0.36, *p* < 0.001, H2a). Teachers’ positive trait emotions were positively related to the expression of naturally felt emotions (*β* = 0.16, *p* < 0.01, H2d), with no correlation with deep acting (*β* = 0.07, *p* = 0.15, H2c) and surface acting (*β* = −0.05, *p* = 0.36, H2b). Moreover, positive trait emotions were positively related to display rules (*β* = 0.52, *p* < 0.001, H2e); however, there was no correlation between negative trait emotions and display rules (*β* = −0.02, *p* = 0.68, H2d).

As for the relationships with the consequences, surface acting was positively correlated with emotional exhaustion (*β* = 0.50, *p* < 0.001, H3a) but negatively correlated with classroom emotional climate (*β* = −0.14, *p* < 0.01, H4a). Deep acting (*β* = 0.29, *p* < 0.001, H4b) and the expression of naturally felt emotions (*β* = 0.38, *p* < 0.001, H4c) were positively correlated with classroom emotional climate but not correlated with emotional exhaustion (*β* = −0.10, *p* = 0.06, H3b; *β* = 0.07, *p* = 0.18, H3c).

### 3.3. Mediating analysis

The correlation of the variables provided evidence for the mediation effects of display rules. In this study, 5,000 bootstrapping samples were generated, and Table 3 lists the standardized estimates of the total, direct, and indirect effects and the 95% confidence interval.

**Table 3:** Standard direct, indirect, and total effects of the 95% confidence intervals.

Independent variable	Dependent variable	Mediator	Direct effect	Indirect effect	95% confidence interval		Total effect
					Lower	Upper	
Positive trait emotions	Deep acting	Display rules	0.07	0.33^***^	0.24	0.42	0.39^***^
Positive trait emotions	Expression of naturally felt emotions	Display rules	0.16^**^	0.29^***^	0.21	0.39	0.45^***^

Bootstrap samples = 5,000; ^**^p < 0.01, ^***^p < 0.001.

Display rules completely mediated positive trait emotions and deep acting; the indirect effect was significant (*β* = 0.33, *p* < 0.001) and the direct effect was not significant (*β* = 0.07, *p* = 0.15). Furthermore, display rules partly mediated positive emotions and the expression of naturally felt emotions. The direct and indirect effects were significant (*β_Direct effect_* = 0.16, *p* < 0.01; *β_Indirect effect_* = 0.29, *p* < 0.001).

## 4. Discussion

The classroom is an emotional setting where TEL is significant for teachers’ wellbeing and students’ learning (Isenbarger and Zembylas, 2006; Yin et al., 2017; Burić and Frenzel, 2021; Xie et al., 2022). However, compared with TEL in generic professional settings, the emotional requirements emphasize the pedagogical functions of TEL more, emotional regulation mainly focuses on solving teacher–student and teacher–self conflicts, and emotional performance tends to be more restrained. These features and previous studies indicate that TEL in classrooms considered both teachers’ emotions and classroom teaching. Therefore, research on its mechanisms would be helpful to clarify how TEL interacts with teaching in class. In the study, Grandey’s integrative model of emotional labor was adopted as the theoretical framework to explore the relationships between the TEL strategies and situation cues (display rules), individual factors (teachers’ trait emotions), individual wellbeing (emotional exhaustion), and organizational performance (classroom emotional climate) in classroom contexts. These variables used to construct the model that are good examples of indicators that can explain the effects of situations and individuals and can contribute to improving teachers’ wellbeing and teaching environment. Results show that some hypotheses were confirmed, and the classroom mechanism of TEL was well explained.

### 4.1. Effects of display rules on TEL strategies in classrooms

Consistent with the studies of Brotheridge and Lee (2003) and Trougakos et al. (2011), results show that display rules were positively related to teachers’ use of deep acting and the expression of naturally felt emotions. In contrast, a positive correlation between display rules and surface acting was not found. The findings fit the self-identity theory that employees who identify with their job role will share the organizational values or goals and are highly likely to use beneficial strategies (Schaubroeck and Jones, 2000).

Another explanation to the relationships between display rules and TEL strategies might be that the display rules varied in different situations and were related to emotional labor in different ways (Grandey and Gabriel, 2015). For instance, positive display rules were linked to deep acting, while negative display rules elicited greater surface acting (Kammeyer-Mueller et al., 2013). In secondary school, students may not be sufficiently mature; therefore, their teachers must exhibit positive emotions while teaching (Hargreaves, 2000; Yin and Lee, 2012). Thus, teachers’ positive understanding of their identity leads them to behave according to more positive display rules and perform deep acting and the expression of naturally felt emotions rather than dissembling or suppressing emotions.

The relationship between display rules and emotional labor strategies indicates that the display rules of a group can be measured in a specific context (Grandey and Melloy, 2017), and it supports the importance of teachers’ perception of display rules in instructing TEL (Yin and Lee, 2012; Stark and Bettini, 2021).

### 4.2. Effects of teachers’ trait emotions on TEL strategies in classrooms

Consistent with the findings of Lee et al. (2016) and Burić et al. (2019), teachers’ negative trait emotions were positively related to surface acting, as the strategy can easily capture negative mood (Grandey and Gabriel, 2015). However, inconsistent with previous studies (Lee et al., 2016; Burić et al., 2019), positive trait emotions were not directly related to deep acting. However, an indirect relationship was found in this study with the mediation of display rules; that is, when teachers’ feelings and required emotions are inconsistent, emotions of love, caring, and enjoyment motivate teachers to perceive display rules as a means to change their cognition, subsequently, inspire their use of deep acting, which is confounded with motivation. (Grandey and Gabriel, 2015).

Positive trait emotions were also positively related to the expression of naturally felt emotions, directly and indirectly, with the mediation of display rules. The correlation is because positive trait emotions motivate teachers to perceive positive display rules and express these positive feelings, which are all beneficial to teacher–student relationships and teachers’ wellbeing. Display rules as mediating variables between positive trait emotions and strategies of deep acting and the expression of naturally felt emotions can be explained from the person-job congruence perspective (Dahling and Johnson, 2013). This perspective proposes that emotional regulation can be predicted by the congruence of an individual’s traits and positive emotional requirements. Therefore, teachers with higher positive trait emotions tend to engage in deep acting and show genuine feelings. In contrast, surface acting is more likely related to negative trait emotions (Diefendorff et al., 2005; Kammeyer-Mueller et al., 2013).

### 4.3. Effects of TEL strategies in classrooms on emotional exhaustion

Consistent with previous studies (Zhang and Zhu, 2008; Näring et al., 2012; Yao et al., 2015; Zhu et al., 2021), surface acting was positively related to emotional exhaustion, possibly because surface acting consumes teachers’ resources according to the conservation of resources theory. Deep acting was not significantly related to emotional exhaustion, consistent with previous studies (Noor and Zainuddin, 2011; Näring et al., 2012). However, other studies (Zhang and Zhu, 2008; Yao et al., 2015) showed that they were negatively related, while another study shows that they were positively related (Zhu et al., 2021). The diverse correlations may be caused by the classroom setting where achieving teaching goals is essential. Limited teaching time requires teachers to adjust their focus from emotional events to instruction. Concentrating on teaching may stop the loss of resources instantly, avoiding emotional exhaustion. In addition, consistent with a previous study (Zhang and Zhu, 2008), the expression of naturally felt emotions did not correlate with emotional exhaustion because using this strategy does not consume too much resources. Furthermore, the null relationship may be related to the increase in teachers’ resources stemming from students’ active participation, love, and respect in classrooms. These reactions provide a sense of value to teachers and a chance to recover energy consumed in deep acting (Duke et al., 2009).

The results can also be explained by the cognitive dissonance theory, which argues that ignoring one’s feelings can cause self-alienation, causing health issues (Grandey and Gabriel, 2015). In the process, surface acting maintains dissonance between expression and feelings, while deep acting attempts to render teachers’ feelings consistent with the required emotions (Hochschild, 1983). Consequently, surface acting is positively related to emotional dissonance, and deep acting is unrelated (Hülsheger and Schewe, 2011). Contrastingly, the expression of naturally felt emotions strategy does not involve emotional dissonance and is unrelated to emotional exhaustion.

### 4.4. Effects of TEL strategies in classrooms on classroom emotional climate

Surface acting is negatively related to classroom emotional climate, while deep acting and the expression of naturally felt emotions are positively related to classroom emotional climate, meaning that in classrooms TEL strategies affect organizational performance. The results are consistent with Hülsheger and Schewe’s (2011) study in which deep acting had positive associations with organizational performance, whereas surface acting had adverse effects. The findings provide supportive evidence to previous research that teachers view their emotions and emotional labor strategies as central determinants of classroom emotional climate (Shewark et al., 2018), and that TEL in classrooms is intertwined into dynamic everyday teaching (Yin and Lee, 2012; Miller and Gkonou, 2018) and contribute to student’s emotional and academic performance (Isenbarger and Zembylas, 2006; Wang et al., 2020; Nyanjom and Naylor, 2021).

The emotion regulation theory can explain the effect of TEL on organizational performance. Viewed as a type of antecedent-focused emotional regulation, deep acting modifies the situation, changing the attention focus, and appraisal of the situation. In comparison, surface acting is response-focused emotional regulation, manipulating emotional expression without cognitive adjusting (Grandey, 2000). In deep acting, teachers’ cognitive judgments affect the display rules of the classroom (Diefendorff et al., 2005) and consequently impact students’ emotional reactions. In contrast, the inauthentic emotions in surface acting can be detected by students and adversely affect classroom performance (Xu, 2018). Moreover, the expression of naturally felt emotions can genuinely deliver teachers’ love and care in a classroom setting through emotional transmission (Frenzel et al., 2018). Therefore, the expression of naturally felt emotions positively relates to the classroom’s emotional climate.

### 4.5. Limitations and future research suggestions

A few limitations of this study must be acknowledged. First, the cross-sectional design could not confirm the direction of causality among the constructs. The integrated process of emotional labor is bidirectional, and feedback loops and cycles exist in this model (Grandey and Gabriel, 2015). For instance, classroom emotional climate may impact TEL strategies and teachers’ trait emotions. Moreover, only a self-report investigation was conducted in this study. Therefore, in future studies, a longitudinal design is needed to clarify the paths better, and qualitative methodologies (e.g., interviews, diaries, and classroom observations) can be utilized (Wang et al., 2020).

Second, though the framework of emotional labor was followed in the study, the variables were not broad enough to cover all aspects. For instance, emotional intelligence can be a vital individual factor that impacts emotional labor (Yin et al., 2013), and a student’s academic performance can be regarded as an organizational consequence caused by TEL (Burić and Frenzel, 2021). Future research is suggested to examine more factors to uncover more facts on the mechanism of emotional labor in classrooms. In addition, compared with TEL in generalized settings, factors in the classroom may be relatively close. Therefore, mediation and regulatory effects remain unclear, which may need further investigation.

Third, influenced by the COVID-19 epidemic, online and offline teaching were both involved in the investigation. As online teaching at home brought teachers with excessive workloads, work–life conflicts, and restricted social interactions, teachers felt more stressed (Carroll et al., 2022) and faced a higher risk of mental disorders (Ghasemi, 2022). Furthermore, the online class involved parents, guardians, and other roles in the classroom, which may impact how teachers manage their emotions; that is, the differences between teachers’ emotional states, emotional requirements, and emotional labor strategies in offline and online classes may lead to the deviation of research results. In future studies, the two types of classroom forms can be studied apart to explore some new features of TEL in classrooms to improve teaching efficiency.

Fourth, the sample was not diverse enough. This study only investigated secondary school teachers and lacked teachers from primary school and university where the emotional requirements and performance may differ (Hargreaves, 2000). The age distribution of the sample (mean age = 37.61 ± 8.87 years) indicates that the sample tends to be young teachers with less length of service. In addition, the sample size can be enlarged with teachers from more regions. Therefore, future research should use larger and a more diversified sample with greater geographical coverage.

Our results also provide some suggestions for teaching practice. First, the positive relationships between positive trait emotions and health benefit strategies (deep acting and the expression of naturally felt emotions) indicate that maintaining teachers’ positive trait emotions is of considerable value. Therefore, measures should be taken to increase positive trait emotions, such as creating a favorable working environment and ensuring higher professional autonomy. Simultaneously, teachers’ negative trait emotions need to be reduced to lessen the consumption of psychological resources (e.g., administrators can reduce unnecessary work and organize upbeat group activities). Second, high deep acting and the expression of naturally felt emotions create a warm classroom climate. Conversely, high surface acting results in an unpleasant emotional climate. This result suggests that training programs can be designed to assist teachers to distinguish positive display rules in classrooms and to improve teachers’ emotional intelligence capability, the ability “to monitor one’s own and other’s feelings and emotions, to discriminate among them, and to use this information to guide one’s thinking and actions” (Salovey and Mayer, 1990, p. 189). With a higher emotional intelligence, teachers can better perceive their own, students’ emotions and the emotional environment, optimize their emotional labor, and promote their pedagogical practices. In addition, as in online classes, more roles (e.g., parents, colleagues, and guardians) have been included, their emotional intelligence capabilities need to be improved as well to inspire teachers with efficient emotional labor.

## 5. Conclusion

This study investigated the mechanism of TEL in classroom settings by Grandey’s integrative model. The results indicate that though similar to previous studies in teachers’ generic professional settings, a few differences exist. For instance, in classrooms, teachers’ positive trait emotions are indirectly related to deep acting, and deep acting and the expression of naturally felt emotions have null correlations with emotional exhaustion. Indeed, this research showed that a positive and caring classroom with a stress-free teacher would be facilitated by assisting teachers in constructing teaching-beneficial display rules, maintaining teachers’ positive trait emotions, and encouraging more frequent use of deep acting and the expression of naturally felt emotions strategies. However, measures to increase teachers’ positive emotions and inspire deep acting and the expression of naturally felt emotions are not discussed enough. In future studies, various variables should be examined in uncovering the mechanism of TEL in classrooms to enhance the quality of class teaching and teachers’ wellbeing, and the studies should use qualitative methodologies, distinguish the online and offline classrooms, and include diverse samples.

## Data availability statement

The original contributions presented in the study are included in the article/supplementary material, further inquiries can be directed to the corresponding author.

## Ethics statement

Written informed consent was obtained from the individual(s) for the publication of any potentially identifiable images or data included in this article.

## Author contributions

PM conceptualized and prepared the materials and wrote the first draft of the manuscript. LZ handled the writing framework, theoretical instruction, and supervision. JY and HD performed the data analysis and writing review. All authors contributed to the study’s conception and design, worked to collect the data, commented on previous versions of the manuscript, and approved the final manuscript.

## Funding

This work was supported by the Humanities and Social Science Fund of the Ministry of Education of China under Grant No. 17YJA880095 and the Fundamental Research Funds for Central Universities under Grant No. 2019TS054.

## Conflict of interest

The authors declare that the research was conducted in the absence of any commercial or financial relationships that could be construed as a potential conflict of interest.

## Publisher’s note

All claims expressed in this article are solely those of the authors and do not necessarily represent those of their affiliated organizations, or those of the publisher, the editors and the reviewers. Any product that may be evaluated in this article, or claim that may be made by its manufacturer, is not guaranteed or endorsed by the publisher.

## Appendix

### Appendix A

**Revised scale of teachers’ emotional labor strategies in the classroom.**

Surface acting:

1. I just pretend to have the emotions I need to display in class.
2. I put on a “mask” to display the emotions I need for teaching in class.
3. The emotions I display to the students in class are not what I feel internally.
4. I hide my real feelings during class teaching.

Deep acting:

1. I make an effort to actually experience the emotions that I need to display during class teaching.
2. Before the class starts, I attempt to truly feel the emotions that teaching needs.
3. When annoyed by students, I try to experience positive feelings by recalling happy moments.
4. I attempt to truly experience the emotions that the class teaching needs.
5. When experiencing anxiety while teaching, I attempt to calm myself down by appreciating the students’ merits.

Expression of naturally felt emotions.

1. The emotions I show to students in class correspond with what I feel spontaneously.
2. It is easy to express my true feelings in class.
3. The emotions I express in class are genuine.
4. The emotions I show during class interactions come naturally.

### Appendix B

**Teacher-perceived classroom emotional climate questionnaire.**

1. Most students react to my emotions.
2. Most students are motivated to learn.
3. Most of the students like my class.
4. The students took an active part in class activities.
5. In class, teaching objectives can always be achieved smoothly and effectively.
6. The classroom climate is mostly relaxed and cheerful.
